# Clinical Consequences of Dental Caries, Parents’ Perception of Child’s Oral Health and Attitudes towards Dental Visits in a Population of 7-Year-Old Children

**DOI:** 10.3390/ijerph18115844

**Published:** 2021-05-29

**Authors:** Justyna Opydo-Szymaczek, Maria Borysewicz-Lewicka, Kinga Andrysiak, Zuzanna Witkowska, Alicja Hoffmann-Przybylska, Piotr Przybylski, Ewa Walicka, Karolina Gerreth

**Affiliations:** 1 Department of Pediatric Dentistry, Chair of Pediatric Dentistry, Poznan University of Medical Sciences, 60-812 Poznan, Poland; jopydo@ump.edu.pl (J.O.-S.); zwitkowska@ump.edu.pl (Z.W.); 2 Department of Risk Group Dentistry, Chair of Pediatric Dentistry, Poznan University of Medical Sciences, 60-812 Poznan, Poland; klstomdz@ump.edu.pl (M.B.-L.); kandrysiak@ump.edu.pl (K.A.); alicjahoffmann@ump.edu.pl (A.H.-P.); piotrprzybylski@ump.edu.pl (P.P.); ewa.nitschke@gmail.com (E.W.)

**Keywords:** dental caries, complications, oral health care, schoolchildren, 7-year-olds

## Abstract

This cross-sectional study aimed to assess the occurrence of the consequences of dental caries and factors affecting dental service utilization in a population of 7-year-old children. The research included oral examination of 7-year-old schoolchildren and socio-medical study of their parents/legal caregivers. It was carried out in five primary schools of two provinces, i.e., Greater Poland and Lubusz. Dental examination was performed in accordance with World Health Organization (WHO) recommendations. The socio-medical study consisted of questionnaires with close-ended questions concerning socioeconomic characteristics of the family, reasons and time of the last visit at the dental office, consequences of child’s oral health problems, parents’ attitude towards dental visits, and parental opinion about their child’s teeth. Factors affecting utilization of dental services were statistically analyzed using univariate logistic regression assuming *p* < 0.05. The pufa index of examined children ranged from 0 to 7 (mean 0.80 ± 1.49), while the dmf index ranged from 0 to 14 (3.86 ± 3.32). Low financial burden of oral health expenditures and university education of at least one of the parents significantly increased the chance of visiting a dentist despite lack of pain (OR = 3.0 and 2.5, respectively). In spite of the availability of free dental care for children, socioeconomic factors still determine the utilization of dental services in Poland. Poor oral health status of examined population and negligence of regular dental check-ups emphasize a need to strengthen oral health literacy of parents and children, promoting proper attitudes towards dental care.

## 1. Introduction

Dental caries (decay) is a multifactorial chronic disease that poses a very serious global health problem since it involves most of the population [1,2,3]. Numerous environmental and individual risk factors are responsible for this disorder’s occurrence and development. These components are related e.g., to the genetic factors, the transmission of germs between hosts, bacterial flora, flow rate and composition of saliva, hygienic and dietary habits, socioeconomic status, parental education, and oral health care availability [3,4,5,6,7,8,9,10,11].

Caries begins with small subsurface demineralization or surface roughness [12]. Such an incipient caries lesion (early enamel caries/white spot lesion) might be treated non-invasively using remineralizing prophylactic agents utilized during daily hygienic procedures and therapy at the dental surgery by professionally applied fluoride treatment [13]. When untreated, the process progresses to a cavitated lesion, and subsequently might involve the pulp giving rise to pain, swelling, and finally systemic signs and symptoms [13]. Therefore, systematic visits to the dental office are necessary. In this way, dental professionals can introduce therapeutic procedures when needed. In effect, they might treat initial (non-cavitated) caries lesions without the bur. On the other hand, more invasive therapy may be used in case of severe carious cavities to avoid local and systemic complications of the disease. Caries and its complications influence the children’s quality of life leading to pain and discomfort, disturbing eating, sleeping, smiling, and affecting school attendance [1,2]. Therefore, periodic visits at the dental office are necessary to diagnose early stages of decay and introduce therapeutic procedures. When the carious process and its complications appear, they affect not only children, but very often their parents and sometimes even the whole families [14]. The problem influences the children’s overall health and quality of life because of pain, discomfort, sleeping and eating difficulties and sometimes school absence [1,2,15].

Unfortunately, dental service utilization among children, including preventive check-ups, is not suitable in many populations [16,17,18,19,20]. According to a cross-sectional, nationwide oral health survey of three-year-olds carried out in Poland in 2002, nearly two-thirds of mothers had never taken their children to a dentist [17]. In a recent study by Mika et al., the mean age of children at their first dental visit was 3.8 years and the most common reason for such visit was pain [19]. The identification of factors affecting the frequency of dental visits and the reasons for them is important in reducing oral health inequalities.

This cross-sectional population-based study aimed to assess the occurrence of the consequences of dental caries, parents’ perception of child’s oral health, and factors affecting dental service utilization in a population of 7-year-old children.

We hypothesized that a high percentage of parents tend to ignore dental caries of children’s primary dentition and the need for regular dental visits, which may lead to serious consequences such as pain, absence at school, difficulties in eating, and dissatisfaction in appearance. The hypothetical factors responsible for parents neglecting children’s oral health were explored in a socio-medical questionnaire.

## 2. Materials and Methods

### 2.1. Ethical Issues

Prior to the study, approval from the Bioethical Committee of the Warsaw Medical University (resolution no. KB/135/2019) and consents from the heads of schools and students’ parents/legal caregivers were obtained.

### 2.2. Study Population

The research was carried out between September 2019 and November 2019 among first-grade students of five primary schools in two western provinces of Poland. The study was a part of the Monitoring of Oral Health and its Determinants, a long-term project, realized since 1997, and funded by the Polish Ministry of Health [21,22].

The selection of schools was done through random sampling technique based on the school listings provided by the Ministry of Education. Five target primary schools were chosen to assure equal representation of inhabitants of rural and urban areas: one school from the city and two schools from villages in Greater Poland Province and two schools situated in an urban-rural commune in Lubusz Province. In each school two classes, out of the whole year, were randomly selected and information about the project presented during the parent-teacher meetings.

Taking into account the number of 7-year-old children in Lubusz and Great Poland Provinces in 2019 equaled 51,713 and 90.5% caries prevalence in this age group, at least 132 participants from both provinces was considered a representative sample size (assuming ±5% error tolerance at 95% confidence interval).

Each parent/legal caregiver was provided with information concerning the examination during the parent-teacher meeting and gave written and informed consent for participation. Full confidentiality of the collected information was provided to the participants of the research.

The study covered dental examination of children and a questionnaire study of their parents/legal caregivers. It was established that during the school year 2019/2020, 236 students aged seven years old attended the aforementioned schools.

Each parent/legal caregiver (136 persons in Greater Poland and 100 in Lubusz) received a questionnaire along with a consent form. After filling in documents at home, they delivered them to the teachers, who subsequently passed them on to the dentists. In total, 87 parents/legal caregivers from Greater Poland and 60 from Lubusz gave consent for their child participation in the dental examination as well as questionnaire study. However 13 sets of dental forms and questionnaires could not be analyzed due to numerous missing data, i.e., five questionnaires were improperly filled out and eight children were absent from school on the day of examination). Therefore, finally, in Greater Poland Province, 80 students and in Lubusz Province 54 children had dental examination performed and their parents/legal caregivers took part in the questionnaire study. 

### 2.3. Dental Examination

Dental examination was performed in accordance with WHO (World Health Organization) recommendations [23]. Children were examined at the school nurse’s office by five dentists with similar clinical experience. Prior to the study, all persons carrying out dental examination participated in special training prepared and performed by the staff (pediatric dentists) of the Warsaw Medical University, which included also calibration process. Teeth assessment was done with the use of a mouth mirror and standard probe (WHO probe) under identical light conditions (artificial light from a headlamp). The procedures were carried out using pictures of different clinical situations and oral examination of 15 patients on two separate occasions, with a one-week interval between sessions. Two days before examination, the training with calibration was repeated at the Chair of Pediatric Dentistry of Poznan University of Medical Sciences by two experienced dentists who are specialists in pediatric dentistry. The credibility of clinical evaluation of the teeth done by examiners was verified based on re-examinations of 20 individuals from the study sample [23]. Substantial intra-examiner and inter-examiner agreements for the diagnosis of dental caries and its consequences were obtained, with Cohen’s κappa values >0.70. 

Prior to the examination, the teeth were not additionally dried or cleaned. For anterior teeth, four surfaces were assessed and recorded, i.e., facial, palatal/lingual, mesial, and distal. For posterior teeth, five surfaces were evaluated and recorded, i.e., occlusal, buccal/facial, lingual, mesial, and distal. Each surface, excluding the inter-proximal surface with no access, of all teeth was assessed. A probe was utilized for verification of visual evidence of caries. A particular tooth was rated and scored as sound, decayed (dt), extracted due to caries (mt), or filled because of caries (ft). The data was utilized to calculate the dmft index, which is the sum of dt, mt, and ft, and expresses dental caries experience. The tooth was considered to have active carious lesion (dt) when the change manifested a detectably soften area, undermined enamel, or unmistakable cavity [23]. Tooth with filling and caries or temporary filling was also assessed as a carious one (dt). The tooth was recorded as missing (mt) when it was extracted because of caries complications (verified by an interview). But when doubts appeared, it was not taken into account. Filled tooth (ft) was defined when there was at least one permanent restoration placed to treat caries. 

Each primary tooth was assessed and scored as sound, decayed (dt), missing (mt) or filled (ft). The scoring system, for each surface of the deciduous tooth, was as follows: A = no caries, B = carious cavity, C = secondary caries, D = filling, E = extracted due to caries. Subsequently, on the basis of clinical examination, the number of teeth with carious cavities (dt), and those extracted (mt) and filled (ft) because of caries were established. A tooth was considered carious when there was a carious cavity or secondary caries seen next to the filling. 

The pufa index for primary dentition was recorded to evaluate the severity of pulpal diseases caused by the untreated carious process. The tooth was rated and scored as having pulpal involvement (p) when the coronal tissues were destroyed by the caries, and only roots and root fragments were left, or the opening of the pulp chamber was visible [18,24]. Ulceration (u) was diagnosed when there was a change of the surrounding soft tissues, e.g., buccal mucosa or tongue, caused by trauma from sharp pieces of a tooth structure, i.e., root fragments or a dislocated tooth with pulpal involvement. Fistula (f) was scored when a pus releasing sinus tract related to a tooth with pulpal involvement was observed. Abscess (a) was diagnosed when a pus containing swelling related to a tooth with pulpal involvement was present. The scoring system, for each deciduous tooth, was as follows: 1 = pulpal involvement, 2 = ulceration, 3 = fistula, 4 = abscess. 

After the oral examination, each child received instructions concerning oral hygiene and diet. All parents/caregivers were provided with data regarding the oral health status of their child, and if he/she had carious lesions or complications of the disease, parents were informed to seek dental services to perform the treatment.

### 2.4. Questionnaire Study of Parents/Legal Caregivers

The socio-medical study was carried out using a questionnaire in Polish. The questionnaire has been previously used in Polish national oral health surveys as a part of the Monitoring of Oral Health and its Determinants [21]. The project follows the guidelines set by the WHO, which recommends the use of simplified structured questionnaires for the collection of data on oral health with items adapted to national specificity [23,25].

Close-ended questions concerned demographic and social characteristics such as: the age of a child, his/her sex, parents’ education (at least one parent with university education/at least one parent with secondary school-leaving examination/neither parent completed secondary school education), place of residence (city/countryside), including also province (Greater Poland/Lubusz), socioeconomic status (in the parent’s opinion—very good/average/poor), perceived burden of oral health care costs on household’s budget (low/medium/heavy financial burden), last visit at the dental office (during last year/more than 12 months ago/two years ago/don’t remember/never been to a dentist), parents neglecting the importance of primary teeth care (“they will fall out anyway”- no/I am not sure/yes), number of children in the family (1/2–5), parental opinion about their child’s teeth (teeth in good and very good condition/teeth in average condition/teeth in poor condition), received information about recommended schedule of visits during last visit at the dental office (yes/no/don’t remember), received detailed information about the condition of child’s teeth during last visit at the dental office (yes/no/don’t remember), and the information whether the child uses private dental service (yes/no).

Finally, 134 sets of questionnaires and results of clinical examination of the students’ teeth were collected. Eighty of 7-year-old schoolchildren (40 children from the city and 40 individuals residing in the village area) were examined in Greater Poland Province. In Lubusz Province 54 children (27 from the city and 27 from the countryside) were included. In Table 1, inclusion and exclusion criteria for 7-year-old individuals and their parents/caregivers in the study are shown, whereas in Table 2, characteristics of the study group and results of the questionnaire study are presented.

In all participants, direct health education program was carried out after teeth evaluation. It included information about oral health and training in tooth brushing. Brochures with information about the principles of the proper oral hygiene, diet and the necessity to visit the dentist as well as the possibility to attend dental visit covered by the National Health Fund were distributed among the respondents (parents/legal caregivers).

### 2.5. Statistical Analysis

The obtained data were statistically analyzed using Statistica software (version 12, StatSoft, Inc. ©, Tulsa, OK, USA, 2014), assuming a statistical significance level of *p* < 0.05. 

The Shapiro-Wilk test indicated the abnormal distribution of the quantitative variables. Thus, they were presented using median and range (minimum-maximum). The values of qualitative variables in groups were compared using the chi-square test. The Yates correction for continuity was used when the observed frequency was small (<5). The univariate logistic regression was used to assess factors contributing to desirable oral health-related behavior (yearly dental check-ups despite lack of pain).

## 3. Results

The pufa index of examined children ranged from 0 to 7 (mean 0.80 ± 1.49), while dmf index ranged from 0 to 14 (3.86 ± 3.32) (Table 3). 

There was statistically significant difference between the percentage of parents rating oral health of children as good and very good in the group with pufa > 0 and pufa = 0 (45.2% and 80.4%, respectively, *p* = 0.000) (Table 4). As many as 45.2% of parents whose children had PUFA > 0 rated their dental health as good or very good.

In the group of children with pufa > 0, there was significantly higher percentage of children reporting oral pain or discomfort during last 12 months (83.3% vs. 45.6%, *p* < 0.0001). Significantly higher percentage of parents from this group were dissatisfied with the child’s teeth appearance (*p* = 0.0144) (Table 5).

The highest percentage of children (33.6%) visited the dental office despite lack of pain or discomfort, similar percentage (30.6%) experienced some pain or discomfort but visited dentist due to other reasons, 16.4% went to the dental office because of pain/discomfort, 12.7% did not report any discomfort and did not appear at the dental office, while 6.7% did not visit a dentist although they suffered due to dental problems (Table 6).

Children who visited the dental office within the last 12 months were further divided into two subgroups. The first subgroup comprised individuals visiting the dental office despite a lack of pain or discomfort. The second group comprised those who experienced pain, absence at school due to pain, and reported pain as a reason for the visit in the past year. In univariate logistic regression, only 4 predictors of yearly dental check-ups without pain were statistically significant: parents’ education level, perceived burden of oral health care costs on household’s budget, parental opinion about child’s oral health and pufa = 0. Good or very good condition of child’s teeth in parents’ opinion, low financial burden of oral health expenditures and university education of at least one of the parents increased significantly the chance of visiting dentist despite lack of pain (OR = 3.5, 3.0, 2.5, and 5.9, respectively). Parents who tended to neglect primary teeth because they fall out anyway had four times lower chance of visiting a dentist without pain (close to significant association *p* = 0.0625) (Table 7).

## 4. Discussion

The research revealed a high mean dmft index in 7-year-old schoolchildren, which amounted to 3.86. This indicates that average 4 teeth in each child were affected by the disease at the time of examination. 

Because of possible complications and consequences of untreated caries, the oral health status of children was described not only with the use of dmft/DMFT index but also by the pufa index presented by Monse et al. in 2010 [5,18,24,26], which evaluates the advanced caries stages with dental sepsis or pulp exposure [5]. It must be remembered that unexplained fever in children might be caused by an abscess associated with carious tooth with infection spreading into the bone of maxilla or mandible, which is a life-threatening complication [12]. The mean pufa index was also high (0.80) in the studied population and the percentage of children with PUFA > 0 was above 30%.

The high percentage of children with complications of dental caries has been observed in developing countries. In India, complications of early childhood caries (pufa > 0) were reported in 71.4% of children between 6 and 72 months of age [24]. In a study conducted in the Philippines, 85% of six-year-old children had a pufa index above zero [18]. In the Iranian population of children aged 6 to 12 years, 25.9% had complications of caries and the mean pufa value was 1.09 [16]. 

Primary teeth caries rates in Poland are lower than in developing countries but higher than in more economically developed parts of European Union [27]. In 2016, national oral health monitoring revealed that 89.4% of 7-year-old children were affected by dental caries with a mean dmft index equal to 5.61 [28]. In the study of Baginska and Rodakowska 72.4% of 7-year-old children living in North-Eastern Poland had at least one tooth affected by caries complications (pufa > 0) [2]. In our study, the percentage of children affected by complications of dental caries and dental caries experience were lower than in the abovementioned studies. However, our study population comes from Western Poland, where the level of socioeconomic status, is relatively high compared to other regions of the country. A recent study carried out in Greater Poland Province revealed that the DMFT of children aged 4 to 7 was equal to 3.4 [11]. Similarly, in the present study, almost four teeth in each child were affected by the disease at the time of examination. Regarding complications of dental caries the percentage of children with pufa > 0 was above 30%, and more than 50% of children suffered from oral pain or discomfort during the last 12 months, which resulted in absence at school in nine individuals. At the same time, only six respondents (4.5%) rated the child’s dentition as poor, which indicates that many caregivers must be unaware of the impact of dental caries as well as the complications of this disease on the oral and general health of their children.

Furthermore, 6.7% of parents/caregivers did not visit a dentist with their child, although he/she suffered due to dental problems. This kind of behavior meets the definition of “child’s dental neglect”, i.e., a failure of caregivers to provide prerequisites of proper oral function via seeking timely dental treatment services necessary to be free from pain and infection [29]. Victims of dental neglect may suffer from dental pain, difficulty eating, sleeping disturbances, low self-esteem, and poor performance at school, affecting overall quality of life [30].

Regular dental visits are considered to be the basis of primary and secondary prevention of oral diseases [31]. The American Dental Association recommends regular preventive dental visits at intervals determined by a dentist based on an individual risk [32]. The American Academy of Pediatric Dentistry recommends the first exam by the age of one year and preventive dental visits every 6 months through adolescence or as indicated by individual risk [33]. Preventive dental visits without pain or discomfort are unique opportunities to establish good cooperation between a dentist and a young patient. However, in our study group, only 33.6% children visited dental office despite lack of pain or discomfort. Factors contributing to this desirable behavior were assessed in univariate logistic regression. It must be emphasized that dental visits for children and some procedures for adults in Poland are free of charge since they are covered by National Health Fund. The scope of dental services is determined by the provisions contained in the Regulation of the Minister of Health of 6 November 2013 on guaranteed services in the field of dental treatment (i.e., Journal of Laws of 2019, item 1199, with amendments) [34]. Parents or legal caregivers of the child have the right to choose a dentist from among those who have signed an agreement with the National Health Fund to provide healthcare services. 

In addition, analysis of the data indicates that education of parents and economic situation of the family significantly affected attitude to preventive visits. Children with at least one parent with a university education had 2.5 times higher chance to regularly visit a dentist despite lack of pain. The low financial burden of oral health care costs on household’s budget was associated with three times higher chance of the regular yearly check-ups. Such observation is in accordance with the results of other researchers [9,35,36,37]. It must be emphasized that the socioeconomic status of the family may impact parents’/caregivers’ perceptions towards the oral health of their children. Individuals with a disadvantaged socioeconomic status might have less access to oral healthcare services because of lower knowledge level regarding dental care needs and the factors associated with caries [9]. Thus, implementation of universal dental care coverage for primary schoolchildren, reduces socioeconomic inequalities in oral health [35,38], but does not eliminate problems related to health literacy of individuals from different economic background. 

In our study, parents of 12 individuals were unaware of the importance of primary dentition, stating that they neglected milk teeth because they would fall out anyway. These parents had four times lower chance of taking their child to a dentist without pain (close to significant association *p* = 0.0625). Unfortunately, caries experience in children is indicative of the disease in the future, and mainly in cases in which individuals are exposed to other risk factors for tooth decay development [9].

The findings of this research have also practical implications for the dental professionals, general doctors and specialists as well as for policymakers. The evaluation of factors affecting dental service utilization is helpful in the organization of public health policies based on scientific evidence [20]. The implementation of universal dental care coverage, mainly for primary schoolchildren, should be encouraged to reduce socioeconomic inequalities in oral health [35,38]. On the other hand, the results of the present paper showed the need for educational program for parents/caregivers as well as children and teachers concerning proper oral health-related habits.

The present study had some strengths and limitations. Firstly, the methodology was properly prepared with international standards according to WHO. Therefore, the results may be easily compared with those obtained by other researchers. Secondly, a comprehensive questionnaire to collect social and socioeconomic data was used. Moreover, adequate examiner reliability measures for caries evaluation were included. The main limitations of the study were the small sample size, analysis of data coming from only two provinces, and a few schools selected for a pilot study, and lack of consent of numerous parents for their child’s dental examination, making the sample not fully representative of general Polish population. Moreover, it would be valuable to include in prospective research on dental caries and its complications, and in analysis of the results information on, e.g., intake of cariogenic and cariostatic foods since such variables might influence observed results. In addition, the caries diagnosis was based only on clinical and not on radiographic findings.

In summary one may say that findings of the present study showed that dental caries constantly affects a large number of primary teeth in schoolchildren in Poland. Notwithstanding the fact that the complications of this disease were seen in numerous individuals, its treatment was very frequently neglected. Therefore, such a situation may influence the quality of life of both the children and their parents/caregivers.

The pilot analysis of factors influencing dental service utilization and attitude to dental visits in the inhabitants of two Polish provinces might be the epidemiologic guidelines for further studies at the country level. It opens future research areas to develop strategies to increase parents’ health awareness and access to pediatric dental care in Poland.

## 5. Conclusions

The present study revealed poor oral health status of the examined population of 7-year-old schoolchildren. Treatment of dental caries was often neglected and its complications were observed and reported by numerous individuals. Results of the study indicate that free dental care for children does not eliminate socioeconomic inequalities in dental services utilization. Economic factors and parents’ education level were important determinants of dental services utilization in examined population. Thus, apart from universal dental care coverage for children, there is a need to strengthen the oral health literacy of parents and children, promoting proper attitudes towards dental care.

## Figures and Tables

**Table 1:** Inclusion and exclusion criteria for 7-year-old individuals and their parents/caregivers in the study.

Inclusion Criteria	Exclusion Criteria
Age between 7 years and 7 years 11 months and 30 days old	Age under 7 years and 8 years old and above
Parental written and informed consent	No parental written and informed consent
Child’s cooperativeness	Child’s uncooperativeness
Subjects from 5 selected schools situated in Greater Poland and Lubusz Provinces	Subjects from other schools than selected prior to study
Subjects residing in Greater Poland and Lubusz Provinces	Subjects from other provinces than Greater Poland and Lubusz
Subjects present at school on days of examination	Subjects absent at school on days of examination
Group with the same ethnic, cultural, demographic, or regional origin	Other ethnic, cultural, demographic, or regional origin
Properly filled in questionnaires returned to examiners	Improperly filled in questionnaires returned to examiners

**Table 2:** Demographics of the study group and results of the questionnaire study.

Patients’ Characteristics		*n* (%) 134 (100.0)
Sex	Female	72 (53.73)
	Male	62 (46.27)
Parents’ education	at least one parent with university education	69 (51.49)
	at least one parent with secondary school-leaving examination	62 (46.27)
	neither parent completed secondary school education	3 (2.24)
Place of residence	City	67 (50.0)
	Countryside	67 (50.0)
Place of residence (province)	Lubusz Province	54 (40.3)
	Greater Poland Province	80 (59.7)
Socioeconomic status (in the parent’s opinion)	very good	22 (16.5)
	average (good)	109 (81.3)
	poor	3 (2.2)
Perceived burden of oral health care costs on household’s budget	low financial burden	32 (23.9)
	medium financial burden	83 (61.9)
	heavy financial burden	19 (14.2)
Last visit at the dental office	during last year	108 (80.6)
	more than 12 months ago	16 (11.9)
	two years ago	1 (0.7)
	don’t remember	1 (0.7)
	never been to a dentist	8 (6.0)
Parents neglecting the importance of primary teeth care (“they will fall out anyway”)	no	120 (89.5)
	I am not sure	4 (3.0)
	yes	10 (7.5)
Number of children in the family	1	25 (18.7)
	2–5	109 (81.3)
Parental opinion about their child’s teeth	teeth in good and very good condition	93 (69.4)
	teeth in average condition	35 (26.1)
	teeth in poor condition	6 (4.5)
Visitors of private dental office	yes	95 (70.9)
	no	39 (29.1)
Received information about recommended schedule of visits during last visit at the dental office *	yes	72 (66.1)
	no	24 (22.0)
	don’t remember	13 (11.9)
Received detailed information about the condition of child’s teeth during last visit at the dental office *	yes	95 (87.2)
	no	8 (7.3)
	don’t remember	6 (5.5)

* Question directed only to parents who took their child to a dentist in the last two years (*n* = 109).

**Table 3:** Dental caries experience and pufa indices in the examined population.

Parameter	Median	Min	Max
pufa	0.00	0	7
d	2.00	0	14
m	0.00	0	3
f	0.00	0	6
dmf	3.50	0	14

**Table 4:** Oral health in examined children as rated by their parents according to pufa.

Oral Health	pufa = 0	pufa > 0	*p*-Value
Poor to average	18 (19.6%)	23 (54.8%)	0.0000
Good and very good	74 (80.4%)	19 (45.2%)	

**Table 5:** Numbers and percentages of subjects reporting oral pain or discomfort, absence at school due to oral pain, difficulty while eating solid foods, problems with smiling, parents’ dissatisfaction with the child’s teeth appearance according to the presence of severely decayed teeth with visible pulpal involvement, ulceration, or abscess (pufa).

Consequences of Oral Health Problems	Answers	pufa > 0 *n* = 42	pufa = 0 *n* = 92	*p*-Value
		*n* (%)	*n* (%)	
Oral pain or discomfort during last 12 months	yes	35 (83.3)	42 (45.6)	0.0000
	no	7 (16.7)	50 (54.4)	
Absence at school due to oral pain	yes	5 (11.9)	4 (4.3)	0.2116
	no	37 (88.1)	88 (95.7)	
Difficulty while eating solid foods	yes	3 (7.1)	8 (8.7)	0.9717
	no	39 (92.9)	84 (91.3)	
Child avoiding smiling because of dental problems	yes	2 (4.8)	0 (0.0)	0.1799
	no	40 (95.2)	92 (100.0)	
Parents’ dissatisfaction with the child’s teeth appearance	yes	21 (50.0)	26 (28.3)	0.0144
	no	21 (50.0)	66 (71.7)	

**Table 6:** Pain as a reason of dental visits.

Was Pain a Reason for the Last Dental Visit? Yes (+)/No (−)	Oral Pain or Discomfort during Last 12 Months Yes (+)/No (−)	Dental Visit during Last 12 Months Yes (+)/No (−)	*n* (%)
			134 (100.0)
+	+	+	22 (16.4)
−	+	+	41 (30.6)
−	−	+	45 (33.6)
−	−	−	17 (12.7)
−	+	−	9 (6.7)

**Table 7:** Odds ratios for desirable oral health-related behavior (yearly dental check-ups despite lack of pain) according to various factors. Only subjects with a dental visit in the past year were included in the analysis (*n* = 108).

Factor	Categories	*n*	OR	95% CI	*p*-Value
Parents’ education	at least one parent with university education	58	2.50	1.13–5.53	0.0225
	neither parent has university education	50	1		
Place of residence	city	55	1.38	0.64–2.96	0.4166
	countryside	53	1		
Place of residence (province)	Lubusz Province	41	0.99	0.45–2.17	1.000
	Greater Poland Province	67	1		
Socioeconomic status (in the parent’s opinion)	very good	19	1.71	0.63–4.64	0.2857
	average and poor	89	1		
Perceived burden of oral health care costs on household’s budget	low financial burden	24	3	1.17–7.67	0.0189
	medium and heavy financial burden	84	1		
Parents neglecting the importance of primary teeth care (“they will fall out anyway”)	no or I am not sure	96	4.06	0.84–19.51	0.0625
	yes	12	1		
Number of children in the family	2–5	87	0.94	0.36–2.47	0.8875
	1	21	1		
Parental opinion about their child’s teeth	teeth in good and very good condition	73	3.47	1.39–8.64	0.0060
	teeth in poor or average condition	35	1		
Visitors of private dental office	yes	81	1.6	0.64–3.98	0.3102
	no	27	1		
Received information about recommended schedule of visits	yes	72	1.69	0.74–3.90	0.2146
	no/don’t remember	36	1		
Received detailed information about the condition of child’s teeth	yes	95	2.64	0.68–10.21	0.2506
	no/don’t remember	13	1		
pufa > 0	−	72	5.91	2.19–15.93	0.0002
	+	36	1		

## Data Availability

The datasets generated for this study are available on request to the corresponding author.

## References

[1] Stephen M.B., Wiedemer J.P., Kushner G.M. (2018). Dental problems in primary care. Am. Fam. Physician.

[2] Bagińska J., Rodakowska E., Wilczyńska-Borawska M., Jamiołkowski J. (2013). Index of clinical consequences of untreated dental caries (pufa) in primary dentition of children from north-east Poland. Adv. Med. Sci..

[3] Gerreth K., Ari T., Bednarz W., Nowicki M., Borysewicz-Lewicka M. (2020). Dental health status and oral health care in nursery school-aged children and their parents living in Poznan (Poland). Med. Princ. Pract..

[4] Rataj-Kulmacz A., Gerreth K., Borysewicz-Lewicka M. (2018). Early childhood caries among a population of nursery-school children from an area with suboptimal fluoride levels in drinking water. Oral Health Prev. Dent..

[5] Baginska J., Stokowska W. (2013). Pulpal involvement-roots-sepsis index: A new method for describing the clinical consequences of untreated dental caries. Med. Princ. Pract..

[6] Chen K.J., Gao S.S., Duangthip D., Lo E.C.M., Chu C.H. (2019). Prevalence of early childhood caries among 5-year-old children: A systematic review. J. Investig. Clin. Dent..

[7] Gerreth K., Zaorska K., Zabel M., Borysewicz-Lewicka M., Nowicki M. (2016). Association of ENAM gene single nucleotide polymorphisms with dental caries in Polish children. Clin. Oral Investig..

[8] Gerreth K., Zaorska K., Zabel M., Borysewicz-Lewicka M., Nowicki M. (2017). Chosen single nucleotide polymorphisms (SNPs) of enamel formation genes and dental caries in a population of Polish children. Adv. Clin. Exp. Med..

[9] Corrêa-Faria P., Paixão-Gonçalves S., Paiva S.M., Pordeus I.A. (2016). Incidence of dental caries in primary dentition and risk factors: A longitudinal study. Braz. Oral Res..

[10] Zaorska K., Szczapa T., Borysewicz-Lewicka M., Nowicki M., Gerreth K. (2021). Prediction of early childhood caries based on single nucleotide polymorphisms using neural networks. Genes.

[11] Opydo-Szymaczek J., Oginska M., Wyrwas B. (2021). Fluoride exposure and factors affecting dental caries in preschool children living in two areas with different natural levels of fluorides. J. Trace Elem. Med. Biol..

[12] Mathur V.P., Dhillon J.K. (2018). Dental caries: A disease which needs attention. Indian J. Pediatr..

[13] Gao S.S., Zhang S., Mei M.L., Lo E.C.-M., Chu C. (2016). Caries remineralisation and arresting effect in children by professionally applied fluoride treatment—A systematic review. BMC Oral Health.

[14] Nishino M., Amarsaikhan B., Furumoto N., Hirao S., Bando H., Nakagawa A., Nomingerel S., Bolorchimeg B., Fujimoto M. (2020). Dental caries in children under five years of age in Mongolia. Int. J. Environ. Res. Public Health.

[15] Bud E.S., Bica C.I., Stoica O.E., Vlasa A., Eșian D., Bucur S.M., Bud A., Chibelean M., Păcurar M. (2021). Observational study regarding the relationship between nutritional status, dental caries, mutans streptococci, and lactobacillus bacterial colonies. Int. J. Environ. Res. Public Health.

[16] Ramazani N., Rezaei S. (2017). Evaluation of the prevalence of clinical consequences of untreated dental caries using PUFA/pufa index in a group of Iranian children. Iran. J. Pediatr..

[17] Szatko F., Wierzbicka M., Dybizbanska E., Struzycka I., Iwanicka-Frankowska E. (2004). Oral health of Polish three-year-olds and mothers’ oral health-related knowledge. Community Dent. Health.

[18] Monse B., Heinrich-Weltzien R., Benzian H., Holmgren C., van Palenstein Helderman W. (2010). PUFA—An index of clinical consequences of untreated dental caries. Community Dent. Oral Epidemiol..

[19] Mika A., Mitus-Kenig M., Zeglen A., Drapella-Gasior D., Rutkowska K., Josko-Ochojska J. (2018). The child’s first dental visit. Age, reasons, oral health status and dental treatment needs among children in Southern Poland. Eur. J. Paediatr. Dent..

[20] Motlagh S.N., Ghasempour S., Bajoulvand R., Hasanvand S., Abbasi-Shakaram S. (2019). Factors affecting demand and utilization of dental services: Evidence from a developing country. Shiraz E Med. J..

[21] Gaszyńska E., Wierzbicka M., Marczak M., Szatko F. (2014). Thirty years of evolution of oral health behaviours and dental caries in urban and rural areas in Poland. Ann. Agric. Environ. Med..

[22] Jodkowska E., Wierzbicka M., Struzycka I., Rusyan E. (2014). Polish public programme of dental caries prevention in children aged 6, 12 and 18 years in 2012. Przegl. Epidemiol..

[23] World Health Organization (WHO) (2013). Oral Health Survey Basic Methods.

[24] Sharna N., Ramakrishnan M., Samuel V., Ravikumar D., Cheenglembi K., Anil S. (2019). Association between early childhood caries and quality of life: Early childhood oral health impact scale and pufa index. Dent. J..

[25] Olczak-Kowalczyk D., Kaczmarek U., Gozdowski D., Turska-Szybka A. (2021). Association of parental-reported vitamin D supplementation with dental caries of 3-year-old children in Poland: A cross-sectional study. Clin. Oral Investig..

[26] Grund K., Goddon I., Schüler I.M., Lehmann T., Heinrich-Weltzien R. (2015). Clinical consequences of untreated dental caries in German 5- and 8-year-olds. BMC Oral Health.

[27] Bencze Z., Mahrouseh N., Andrade C.A.S., Kovács N., Varga O. (2021). The burden of early childhood caries in children under 5 years old in the European Union and associated risk factors: An ecological study. Nutrients.

[28] Polish Ministry of Health, Monitoring the Oral Health of the Polish Population in 2016–2020. Oral Health and Its Determinants in the Polish Population Aged 5, 7 and 12 in 2016, Ministry of Health, 2018. https://www.gov.pl/web/zdrowie/monitorowanie-stanu-zdrowia-jamy-ustnej-populacji-polskiej-w-latach-2016-2020.

[29] Ramazani N. (2014). Child dental neglect: A short review. Int. J. High Risk Behav. Addict..

[30] Lourenco C.B., Saintrain M., Vieira A.P. (2013). Child, neglect and oral health. BMC Pediatr..

[31] Hahn T., Kraus C., Hooper-Lane C. (2017). What is the optimal frequency for dental checkups for children and adults?. J. Fam. Pract..

[32] Giannobile W., Braun T., Caplis A., Doucette-Stamm L., Duff G., Komman S. (2013). Patient stratification for preventive care in dentistry. J. Dent. Res..

[33] American Academy of Pediatric Dentistry (2013). Guideline on periodicity of examination, preventive dental services, anticipatory guidance/counseling, and oral treatment for infants, children, and adolescents. Pediatr. Dent..

[34] Obwieszczenie Ministra Zdrowia z Dnia 30 maja 2019 r. w Sprawie Ogłoszenia Jednolitego Tekstu Rozporządzenia Ministra Zdrowia w Sprawie Świadczeń Gwarantowanych z Zakresu Leczenia Stomatologicznego (Dz. U. z 2019 r. poz. 1199 ze zm.). http://prawo.sejm.gov.pl/isap.nsf/download.xsp/WDU20190001199/O/D20191199.pdf.

[35] Peres M.A., Ju X., Mittinty M., Spencer A.J., Do L.G. (2019). Modifiable factors explain socioeconomic inequalities in children’s dental caries. J. Dent. Res..

[36] Kramer A.C.A., Petzold M., Hakeberg M., Östberg A.L. (2018). Multiple socioeconomic factors and dental caries in swedish children and adolescents. Caries Res..

[37] Elamin A., Garemo M., Gardner A. (2018). Dental caries and their association with socioeconomic characteristics, oral hygiene practices and eating habits among preschool children in Abu Dhabi, United Arab Emirates—The NOPLAS project. BMC Oral Health.

[38] Mathur M.R., Williams D.M., Reddy K.S., Watt R.G. (2015). Universal health coverage: A unique policy opportunity for oral health. J. Dent. Res..

